# A Descriptive Pilot Assessment of Simple Outpatient Functional Measures Following SARS-CoV-2 Infection in Adults with Chronic Diseases

**DOI:** 10.3390/healthcare14142104

**Published:** 2026-07-14

**Authors:** Despina Paula Andrei, Adrian Militaru, Nicolae Ghita, Petru Armean

**Affiliations:** 1 “Carol Davila” University of Medicine and Pharmacy, 020021 Bucharest, Romania; adrian.militaru@drd.umfcd.ro (A.M.); nicolae.ghita@drd.umfcd.ro (N.G.); petru.armean@umfcd.ro (P.A.); 2 Clinical Department of Neurosurgery, “Elias” University Emergency Hospital, 17 Marasti Boulevard, 011461 Bucharest, Romania; 3 Clinical Department of Urology, “St. John” Clinical Hospital, 13 Vitan-Bârzesti Road, Sector 4, 042122 Bucharest, Romania; 4 Clinical Department of Internal Medicine, “Prof. Dr. Theodor Burghele” Clinical Hospital, 20 Panduri Road, 061344 Bucharest, Romania

**Keywords:** symptoms following SARS-CoV-2 infection, chronic diseases, outpatient care, functional impairment, pilot study, functional assessment

## Abstract

**Highlights:**

**What are the main findings?**

- Symptoms reported after SARS-CoV-2 infection, particularly fatigue and dyspnea, were commonly observed among adults with chronic diseases assessed during the post-acute recovery period.
- Simple outpatient functional measures, including the mMRC scale, Borg scale, pulse oximetry, and the 1-min sit-to-stand test, provided a structured description of symptom burden and functional findings within this pilot cohort.

**What are the implications of the main findings?**

- The findings support further investigation of simple outpatient functional assessment approaches for patients during post-acute recovery following SARS-CoV-2 infection.
- These exploratory results may inform future studies evaluating structured assessment strategies and multidisciplinary follow-up in patients with chronic diseases after SARS-CoV-2 infection.

**Abstract:**

**Background**: Persistent functional symptoms may occur during post-acute recovery following SARS-CoV-2 infection, particularly among patients with pre-existing chronic diseases. However, structured outpatient approaches for the systematic assessment of functional status remain insufficiently described. This pilot study aimed to characterize functional findings in adults with chronic diseases assessed after SARS-CoV-2 infection using simple outpatient assessment measures and to explore a descriptive approach for characterizing functional findings in routine outpatient practice. **Methods**: We conducted an observational, cross-sectional pilot study including 38 consecutive adult outpatients (aged 38–82 years) evaluated between April and June 2025 in a university-affiliated outpatient setting. Participants were assessed during the post-acute recovery period, between four weeks and six months after acute SARS-CoV-2 infection. Functional evaluation included the modified Medical Research Council (mMRC) dyspnea scale, Borg scale (at rest and post-effort), pulse oximetry at rest and after a one-minute sit-to-stand test, and heart rate monitoring. Data were analyzed descriptively in accordance with the exploratory nature of the study. **Results**: Fatigue (57.9%; 95% CI: 42.2–72.1) and dyspnea (44.7%; 95% CI: 30.2–60.3) were the most frequently reported symptoms among participants assessed after SARS-CoV-2 infection, followed by sleep disturbances (42.1%; 95% CI: 28.0–57.8), anxiety (36.8%; 95% CI: 23.8–52.4), and palpitations (26.3%; 95% CI: 14.2–43.7). Post-effort oxygen desaturation ≥4% was observed in 11 participants (28.9%), whereas a final post-effort SpO_2_ below 94% was recorded in 22 participants (57.9%). Five participants (13.2%) demonstrated a final post-effort SpO_2_ below 92%, and 10 participants (26.3%) met both the criteria of post-effort desaturation ≥4% and final post-effort SpO_2_ below 94%. Within the exploratory categorization approach, participants were grouped into preliminary mild (28.9%), moderate (36.8%), and severe (34.2%) functional categories. **Conclusions**: This exploratory pilot study describes functional findings observed in adults with chronic diseases during post-acute recovery following SARS-CoV-2 infection. Simple outpatient assessment measures may contribute to a structured description of symptom burden and functional status in routine clinical practice. The proposed categorization should be interpreted as a descriptive exploratory approach rather than as a validated clinical framework or decision-making tool. Further prospective studies involving larger cohorts are needed to evaluate reproducibility, clinical relevance, and potential applications in outpatient assessment.

## 1. Introduction

The coronavirus disease 2019 (COVID-19) pandemic continues to have long-term consequences for many individuals, even several years after acute infection. Symptoms reported following SARS-CoV-2 infection remain common and may affect physical, psychological, cognitive, and social functioning during the post-acute recovery period. According to the World Health Organization (WHO), post-COVID condition generally refers to symptoms that occur in individuals with a history of probable or confirmed SARS-CoV-2 infection, persist for at least two months, and usually appear three months after the initial infection [1,2]. However, persistent manifestations may also be observed during earlier stages of post-acute recovery and may represent clinically relevant challenges requiring ongoing outpatient evaluation. Recent cohort studies have identified fatigue, dyspnea, palpitations, sleep disturbances, and anxiety among the most frequently reported persistent symptoms, with some manifestations persisting for many months after the acute phase of infection [3,4,5,6].

Patients with pre-existing chronic diseases, including hypertension, diabetes mellitus, chronic obstructive pulmonary disease (COPD), ischemic heart disease, and cancer, may represent a particularly vulnerable population. The impact of these conditions may vary substantially according to disease severity, physiological reserve, and multimorbidity burden. Chronic diseases and multimorbidity may contribute to reduced physiological reserve and greater vulnerability to functional decline following acute illness [7]. At the same time, interpretation of persistent symptoms remains challenging because manifestations such as fatigue, dyspnea, exercise intolerance, and sleep disturbances may also reflect the underlying chronic disease burden rather than being exclusively attributable to SARS-CoV-2 infection.

International recommendations increasingly emphasize the importance of structured assessment and rehabilitation approaches for individuals experiencing persistent symptoms following SARS-CoV-2 infection. Existing recommendations, including those issued by the National Institute for Health and Care Excellence (NICE), the European Respiratory Society (ERS), and pulmonary rehabilitation programs, support multidimensional evaluation of patients presenting with ongoing symptoms after COVID-19 [8,9,10]. Nevertheless, many existing approaches focus primarily on respiratory recovery or rehabilitation principles and provide limited practical guidance regarding the outpatient assessment of heterogeneous populations with multiple chronic diseases.

Within this context, it is important to distinguish between functional assessment and rehabilitation referral. Functional assessment aims to identify persistent symptoms and objective functional findings using standardized clinical tools, whereas rehabilitation referral represents a subsequent clinical decision based on the overall evaluation. Although related, these processes address different stages of patient management and should not be considered interchangeable.

Simple clinical instruments such as the modified Medical Research Council dyspnea scale, Borg perceived exertion scale, pulse oximetry, and the one-minute sit-to-stand test have been widely used to assess dyspnea, perceived effort, exercise tolerance, and physiological responses in respiratory rehabilitation and post-acute recovery populations [9,11].

These measures may contribute to a more systematic description of symptom burden and functional status in routine outpatient practice.

Although rehabilitation services for individuals recovering from COVID-19 have gradually become available internationally, published outpatient assessment approaches specifically addressing adults with chronic diseases following SARS-CoV-2 infection appear to remain relatively limited within the Romanian literature and clinical context. Existing national guidance documents provide broad recommendations regarding rehabilitation and chronic disease management, although some rehabilitation and chronic disease management recommendations are available. However, standardized outpatient assessment approaches specifically adapted to heterogeneous populations of adults with chronic diseases assessed after SARS-CoV-2 infection remain insufficiently described. Consequently, the integration of simple functional assessment measures into routine outpatient care remains an area requiring further investigation [10,12].

Therefore, the present study was designed as an exploratory pilot study describing functional findings in adults with chronic diseases evaluated after SARS-CoV-2 infection using simple outpatient assessment measures. The primary objectives were:

(1) To identify the most frequently reported symptoms among patients with chronic diseases assessed after SARS-CoV-2 infection;
(2) To describe functional findings obtained using simple outpatient clinical assessment tools, including the mMRC dyspnea scale, Borg scale, pulse oximetry, heart rate monitoring, and the one-minute sit-to-stand test;
(3) To explore whether these measures could contribute to a more structured description of functional status in routine outpatient practice and inform future research in this field.

## 2. Materials and Methods

### 2.1. Study Design and Participants

This observational cross-sectional study was designed as an exploratory pilot investigation aimed at describing functional findings in individuals with chronic diseases assessed after SARS-CoV-2 infection using simple outpatient clinical assessment measures.

A total of 38 consecutive adult outpatients (aged 38–82 years) were prospectively recruited between April and June 2025 from specialist outpatient clinics within the university-affiliated “Prof. Dr. Theodor Burghele” Clinical Hospital, Bucharest, Romania.

Consecutive recruitment referred to inclusion according to the order in which eligible patients presented during routine outpatient visits and agreed to participate, without additional geographic or socioeconomic selection criteria. The number of screened patients and refusal rates were not systematically recorded because the study was designed as an exploratory pilot investigation conducted within routine outpatient practice.

Eligible participants had documented evidence of at least one chronic condition, including hypertension, diabetes mellitus, chronic obstructive pulmonary disease (COPD), ischemic heart disease, or cancer, together with laboratory-confirmed SARS-CoV-2 infection (PCR or antigen testing). Cancer was included because malignant diseases represent chronic conditions frequently associated with reduced physiological reserve and increased vulnerability to functional decline following acute illness.

Participants were evaluated after recovery from the acute phase of infection between four weeks and six months after SARS-CoV-2 infection. This interval was selected to explore functional manifestations during the post-acute recovery period rather than to apply the WHO definition of post-COVID condition exclusively. Information regarding acute COVID-19 severity, hospitalization history, oxygen supplementation during the acute phase, vaccination status, previous rehabilitation interventions, and previous SARS-CoV-2 reinfections was not systematically collected because these variables were outside the scope of the initial pilot study design.

Patients were included regardless of the initial severity of COVID-19 infection, provided they were clinically stable at the time of assessment.

Exclusion criteria comprised:

- Acute cardiac or pulmonary exacerbations;
- Decompensated chronic disease;
- Severe clinical instability preventing safe functional assessment;
- Absence of laboratory-confirmed SARS-CoV-2 infection;
- Neurological or psychiatric disorders impairing communication or participation;
- Inability to safely perform functional assessment procedures.

No formal sample size calculation was performed because the study was designed as an exploratory pilot investigation focused primarily on describing symptom patterns and functional findings within a real-world outpatient setting rather than on hypothesis testing.

Therefore, the sample size was pragmatically determined according to the number of eligible consecutive patients presenting during the study period.

Given the single-center design and consecutive sampling strategy, the study population was not intended to be representative of the general population but rather to reflect routine outpatient practice in a tertiary-care setting. Because recruitment was performed within a tertiary-care outpatient setting, the study population may include patients with more complex clinical profiles than those encountered in primary care or community settings.

The study was conducted and reported according to the Strengthening the Reporting of Observational Studies in Epidemiology (STROBE) guidelines.

### 2.2. Ethical and Data Protection Considerations

The study was conducted in accordance with the principles of the Declaration of Helsinki and applicable Romanian legislation, including Law No. 190/2018 and the European Union General Data Protection Regulation (GDPR) 2016/679 [13].

Written informed consent was obtained from all participants prior to study inclusion following detailed explanation of the study procedures, confidentiality safeguards, and the voluntary nature of participation.

Medical history interviews performed during outpatient consultations were audio-recorded exclusively for research documentation and data verification purposes, with explicit participant consent. The recordings were used solely to verify the accuracy and completeness of information entered into the study database and were not transcribed or analyzed using qualitative research methods. Access to the recordings was restricted to the principal investigator. All recordings were anonymized, securely stored in accordance with institutional data protection requirements, and permanently deleted after completion of the data verification process. The recording procedure was included within the approved study protocol and covered by the informed consent process.

Participant confidentiality was strictly maintained, and all collected information was securely stored in accordance with institutional policies and legal requirements.

The study protocol was approved by the Ethics Committee of “Prof. Dr. Theodor Burghele” Clinical Hospital, Bucharest (Approval No. 4/12 October 2020). The study formed part of an ongoing doctoral research project involving continuous patient recruitment and no substantial protocol modifications requiring additional ethical approval. Continuation of the ethical authorization was formally requested and granted for the extended research period. Because no changes affecting study objectives, participant risk, informed consent procedures, audio-recording procedures, functional assessment measures, or data protection safeguards were introduced, the Ethics Committee considered the original protocol and its continuation valid throughout the study period.

### 2.3. Clinical and Functional Assessment

Participants completed a standardized clinical assessment form developed for the evaluation of individuals assessed after SARS-CoV-2 infection, including:

- Age;
- Sex;
- Place of residence;
- Chronic disease type;
- Time since SARS-CoV-2 infection;
- Relevant medical history.

Variables such as body mass index, smoking history, medication regimens, and disease-specific severity indicators (e.g., FEV1, HbA1c, NYHA class, cancer stage) were not systematically available within this exploratory pilot investigation.

Five commonly reported persistent symptoms potentially associated with functional limitation were systematically assessed:

- Fatigue;
- Dyspnea;
- Palpitations;
- Sleep disturbances;
- Anxiety.

The presence of fatigue, palpitations, sleep disturbances, and anxiety was assessed using a standardized symptom checklist administered during the outpatient consultation. Symptoms were recorded as present or absent according to participant self-report at the time of assessment and documented by the investigator using the standardized clinical assessment form. No validated symptom-specific instruments were used to quantify the severity of anxiety, sleep disturbances, fatigue, or palpitations because the objective of this exploratory pilot study was to describe the occurrence of commonly reported symptoms rather than to perform detailed psychometric or symptom-severity assessment. Dyspnea was additionally evaluated using the modified Medical Research Council (mMRC) dyspnea scale.

Functional evaluation incorporated subjective symptom scales and objective physiological measurements:

- Modified Medical Research Council (mMRC) dyspnea scale (0–4) [14];
- Borg perceived effort scale (0–10), recorded at rest and immediately after effort [15];
- One-minute sit-to-stand test (1-min STS) [16,17];
- Peripheral oxygen saturation (SpO_2_);
- Heart rate measurements before and after the 1-min STS test [18].

The one-minute sit-to-stand (1-min STS) test was performed using a standard outpatient chair with armrests and an approximate seat height of 45 cm. Participants remained seated for at least 5 min before baseline measurements of heart rate and peripheral oxygen saturation (SpO_2_) were obtained. During testing, participants were permitted to use the armrests for support when necessary for safety and comfort.

Heart rate and SpO_2_ measurements were repeated immediately after completion of the test. All assessments were conducted under direct investigator supervision, and participants were continuously monitored for symptoms and physiological responses throughout the procedure. Predefined safety criteria for test termination included severe dyspnea, chest pain, or dizziness. However, no participant met these criteria or required premature termination of the test. Patients receiving chronic supplemental oxygen therapy were not included in the study.

Pulse oximetry measurements were obtained using a Contec CMS50D fingertip pulse oximeter (Contec Medical Systems Co., Ltd., Qinhuangdao, China). Pulse oximetry measurements were performed under routine outpatient conditions using the same device throughout the study period. Resting measurements were obtained after participants had remained seated for at least 5 min. Oxygen saturation values were recorded only after a stable signal and reading had been achieved. If signal instability occurred, measurements were repeated until a stable value was obtained. Obvious factors potentially affecting measurement quality, including inappropriate sensor positioning, were corrected before recording values. Post-exertional SpO_2_ measurements were obtained immediately after completion of the 1-min STS test. No formal calibration procedures beyond routine clinical use of the device were performed.

To reduce potential interobserver variability, all functional assessments were conducted by a single investigator using a predefined standardized protocol and identical assessment criteria throughout the study period, thereby ensuring methodological consistency across all evaluations.

The selected instruments are standardized clinical assessment tools commonly used in respiratory rehabilitation and post-acute recovery assessment following SARS-CoV-2 infection; therefore, internal consistency measures such as Cronbach’s alpha were not considered applicable.

### 2.4. Development of an Exploratory Functional Categorization Approach

An exploratory functional categorization approach was developed pragmatically using published rehabilitation recommendations following SARS-CoV-2 infection and commonly used outpatient functional assessment measures. The intention was not to establish or validate a clinical framework or decision rule, but rather to explore whether simple clinical indicators could contribute to a more structured description of functional status in routine outpatient practice.

Threshold values were selected pragmatically based on commonly reported clinical indicators of dyspnea severity, exertional oxygen desaturation, and post-effort oxygen saturation described in the outpatient rehabilitation and functional assessment literature [19,20]. These thresholds were not intended to represent validated cut-offs for mixed chronic disease outpatients assessed after SARS-CoV-2 infection. Instead, they were used solely for descriptive and exploratory purposes to organize observed symptom burden and physiological findings within this pilot cohort. Therefore, the resulting categories should be interpreted as a pragmatic descriptive classification rather than as validated severity strata.

### 2.5. Preliminary Functional Categorization

For descriptive and exploratory purposes, participants were grouped into three preliminary functional categories according to combined symptom severity and physiological findings:

**Mild category**

- mMRC < 2;
- SpO_2_ decrease < 4%;
- Final SpO_2_ ≥ 94%.

**Moderate category**

- mMRC ≥ 2 or SpO_2_ decrease ≥ 4%;
- Final SpO_2_ ≥ 92%.

**Severe category**

- mMRC ≥ 3 and/or final SpO_2_ < 92%.

Within the severe category, findings were further interpreted descriptively according to the dominant feature. Participants meeting the severe criterion primarily through mMRC ≥ 3 were considered to have symptom-dominant severe functional limitation, whereas those meeting the severe criterion through final post-effort SpO_2_ < 92% were considered to have physiological desaturation-dominant severe findings. This distinction was used for interpretative purposes only and was not intended to define separate validated clinical subgroups.

To ensure that all participants could be assigned to a category, classification was performed hierarchically. Participants meeting any criterion for the severe category were classified as severe. Participants not meeting severe criteria but presenting either mMRC ≥ 2, exertional SpO_2_ decrease ≥ 4%, or a final post-effort SpO_2_ between 92% and 93% were classified as moderate. Remaining participants were classified as mild. These rules were applied solely for descriptive purposes within the exploratory categorization approach.

Additional symptoms including fatigue, anxiety, and palpitations were considered complementary clinical observations within the exploratory assessment process. These findings were incorporated as complementary clinical observations within the exploratory categorization approach and were not intended to establish causal attribution specifically to SARS-CoV-2 infection, given the potential overlap with manifestations of pre-existing chronic diseases and other contributing factors.

### 2.6. Data Management and Statistical Analysis

Clinical information was entered into a structured electronic database designed specifically for the study. Descriptive statistical analyses were performed using Microsoft Excel for Microsoft 365 (Version 16.94; Microsoft Corporation, Redmond, WA, USA).

All enrolled participants completed the planned assessment procedures. No missing data were identified for the variables included in the descriptive analyses.

Statistical analysis was primarily descriptive and aimed to characterize symptom patterns and functional findings within the study cohort.

Categorical variables are presented as frequencies and percentages, whereas continuous variables are summarized as means ± standard deviations. For descriptive purposes, 95% confidence intervals (95% CIs) were additionally calculated for the main symptom frequencies to provide an estimate of the precision of the observed proportions.

Given the exploratory pilot design and limited sample size, inferential statistical analyses and formal normality testing were not performed because the study was not designed or powered for hypothesis testing. Descriptive subgroup comparisons according to chronic disease categories were considered exploratory and should be interpreted with caution, as the limited sample size did not permit reliable disease-specific analyses.

## 3. Results

A total of 38 adult outpatients assessed after SARS-CoV-2 infection (mean age 64.2 ± 13.2 years, range 38–82 years) were included in the study. The majority were male (55.3%), while 44.7% were female. Most participants had at least one chronic condition, predominantly hypertension (60.5%), followed by ischemic heart disease (34.2%), diabetes mellitus (23.7%), chronic obstructive pulmonary disease (21.1%), and cancer (5.3%).

The baseline characteristics of the study population are summarized in Table 1.

Time since SARS-CoV-2 infection varied across participants. Six patients (15.8%) were evaluated between 4 and <8 weeks after infection, 10 patients (26.3%) between 8 and <12 weeks, and 22 patients (57.9%) between 12 weeks and 6 months after infection (Table 2). Most participants were therefore assessed beyond the early post-acute recovery phase. Nevertheless, because a subset of participants was evaluated before the three-month threshold proposed by the World Health Organization for post-COVID condition, part of the study population may represent prolonged post-acute recovery rather than established post-COVID condition according to WHO criteria.

### 3.1. Symptoms Reported Following SARS-CoV-2 Infection

Fatigue was reported by 22 of 38 participants (57.9%; 95% CI: 42.2–72.1) and dyspnea by 17 of 38 participants (44.7%; 95% CI: 30.2–60.3). Other commonly reported symptoms included sleep disturbances in 16 participants (42.1%; 95% CI: 28.0–57.8), anxiety in 14 participants (36.8%; 95% CI: 23.8–52.4), and palpitations in 10 participants (26.3%; 95% CI: 14.2–43.7).

### 3.2. Functional Assessment Findings

The mean mMRC dyspnea score was 1.1 ± 0.9, suggesting generally mild-to-moderate levels of perceived activity limitation within the cohort. Participants completed a mean of 30.7 ± 4.1 repetitions during the one-minute sit-to-stand test (range: 25–39 repetitions).

The mean Borg score increased from 0.8 ± 0.7 at rest to 4.5 ± 1.6 following the one-minute sit-to-stand test.

Mean oxygen saturation (SpO_2_) decreased from 96.0% ± 1.9 at rest to 93.2% ± 2.4 following effort, while mean heart rate increased from 80.2 ± 10.4 bpm at rest to 105.6 ± 12.7 bpm after testing.

The principal functional assessment findings are summarized in Table 3.

Post-effort oxygen desaturation ≥4% was observed in 11 participants (28.9%), whereas a final post-effort SpO_2_ below 94% was recorded in 22 participants (57.9%). Five participants (13.2%) demonstrated a final post-effort SpO_2_ below 92%, corresponding to the threshold used within the exploratory severe functional category. Ten participants (26.3%) met both criteria of post-effort desaturation ≥4% and final post-effort SpO_2_ below 94%.

To further clarify the composition of the exploratory severe functional category, a descriptive cross-tabulation was performed (Table 4). Among the 13 participants classified as severe, 8 met the severe criterion through mMRC ≥ 3 alone, 2 through final post-effort SpO_2_ <92% alone, and 3 through both criteria. These findings indicate that severe category assignment reflected both symptom-dominant and physiological desaturation-dominant patterns rather than elevated dyspnea scores alone.

### 3.3. Distribution of Participants According to the Exploratory Functional Categorization

For descriptive and exploratory purposes, participants were grouped into preliminary functional categories based on the predefined categorization approach described in Section 2 (Table 5).

Within this exploratory categorization, 28.9% of participants were classified in the mild category, 36.8% in the moderate category, and 34.2% in the severe category.

### 3.4. Exploratory Observations of Functional Findings

Within this exploratory pilot study, patterns of functional findings were used to describe differences in symptom burden and physiological responses observed across participants.

Individuals with milder functional findings generally demonstrated lower symptom burden and minimal post-effort desaturation, whereas participants presenting with greater dyspnea severity or oxygen desaturation were more frequently represented within the moderate and severe functional categories.

Because symptoms such as dyspnea, fatigue, and exercise intolerance may also reflect underlying chronic disease burden, these observations should not be interpreted as evidence of causal attribution specifically to SARS-CoV-2 infection.

The assessment process was based on simple outpatient clinical measures that were readily integrated into routine clinical evaluation. However, assessment duration was not systematically recorded, and therefore no formal conclusions regarding feasibility can be drawn from the present study.

The exploratory categorization approach used in this pilot investigation is summarized in Table 5.

## 4. Discussion

This exploratory pilot study describes symptoms and functional findings observed in adults with chronic diseases assessed after SARS-CoV-2 infection using simple outpatient clinical measures. Fatigue and dyspnea were the most frequently reported symptoms in the present cohort. However, given the absence of pre-infection functional data and a comparator group, these findings should be interpreted as symptoms reported after SARS-CoV-2 infection rather than manifestations that can be directly attributed to the infection itself. Similar findings have been reported in recent longitudinal studies, where fatigue, post-effort dyspnea, and reduced exercise tolerance were identified among the most persistent manifestations affecting daily functioning and quality of life [3,21,22,23,24,25,26].

The interpretation of symptoms reported after SARS-CoV-2 infection in patients with chronic diseases requires careful consideration because several manifestations frequently reported after SARS-CoV-2 infection may also reflect pre-existing disease burden. Symptoms such as dyspnea, fatigue, and exercise intolerance may already be present in patients with chronic obstructive pulmonary disease, ischemic heart disease, or advanced metabolic disorders independent of SARS-CoV-2 infection. Consequently, distinguishing between functional manifestations potentially associated with SARS-CoV-2 infection and pre-existing functional limitations remains challenging. Because of the exploratory design, limited sample size, and heterogeneity of the cohort, disease-specific conclusions cannot be drawn from the present findings.

The predominance of hypertension within the study population also deserves consideration. Chronic diseases included in the present study may differ substantially in their potential impact on functional status, exercise tolerance, and physiological responses. However, because disease-specific severity indicators, multimorbidity patterns, and detailed subgroup analyses were not available within this exploratory pilot investigation, disease-specific interpretations should be avoided. Future studies involving larger cohorts should examine the contribution of individual chronic disease profiles and multimorbidity burden to functional findings observed after SARS-CoV-2 infection. Functional vulnerability is likely influenced not only by diagnostic category but also by disease severity, physiological reserve, and the presence of multiple comorbidities. Consequently, future investigations should consider stratification according to disease severity and cumulative comorbidity burden. An additional consideration is the absence of systematically collected information regarding acute COVID-19 severity, hospitalization history, oxygen supplementation, vaccination status, previous rehabilitation interventions, and SARS-CoV-2 reinfections. These factors may substantially influence symptom persistence and functional recovery trajectories. Consequently, the functional findings observed in the present study should be interpreted descriptively, as the potential contribution of acute disease severity and previous clinical course could not be evaluated. In addition, several potentially relevant clinical variables, including body mass index, smoking status, multimorbidity burden, prior rehabilitation interventions, vaccination history, and baseline functional status before SARS-CoV-2 infection, were not systematically available within the study dataset. The absence of these variables limits the ability to explore factors potentially associated with the observed functional findings and requires cautious interpretation of the results. Psychological and psychosomatic mechanisms may also contribute to persistent symptom perception after COVID-19. Fatigue, sleep disturbances, anxiety, and reduced exercise tolerance may result from complex interactions involving physiological recovery, emotional distress, chronic disease burden, and behavioral adaptations following prolonged illness [23,24]. Previous studies have demonstrated that psychological factors frequently coexist with physical symptoms in individuals reporting persistent symptoms after SARS-CoV-2 infection and may substantially influence perceived functional status and quality of life [23,27]. Therefore, interpretation of persistent symptoms should extend beyond isolated physiological measures and incorporate broader multidimensional evaluation.

Functional assessment in outpatient settings remains clinically relevant because many patients continue to report persistent symptoms despite the absence of severe physiological abnormalities. Simple tools such as the modified Medical Research Council dyspnea scale, Borg perceived exertion scale, pulse oximetry, and the one-minute sit-to-stand test are attractive because they are inexpensive, accessible, relatively easy to implement in routine outpatient practice, and have demonstrated utility in adult respiratory and post-acute recovery populations [16,17,28,29,30]. Existing pulmonary and cardiac rehabilitation pathways already incorporate combinations of symptom assessment and functional evaluation to support patient management [9,19,20,31]. However, these approaches are generally directed toward specific clinical populations and may provide limited guidance for heterogeneous outpatient populations with multiple chronic diseases. The exploratory categorization approach used in the present study was intended only to examine whether simple functional indicators could contribute to a more structured description of functional status in routine outpatient practice rather than to establish a validated clinical framework or decision-making model.

It should also be acknowledged that the severe category may include clinically different patterns of impairment. Some participants may be classified as severe because of pronounced dyspnea without marked oxygen desaturation, whereas others may meet severe criteria primarily because of post-effort SpO_2_ < 92%. These patterns may reflect different clinical phenotypes, including symptom-dominant limitation and physiological desaturation-dominant impairment. Therefore, the severe category should be interpreted as a broad exploratory descriptor rather than as a homogeneous clinical phenotype.

To our knowledge, published outpatient functional assessment approaches specifically addressing adults with chronic diseases assessed after SARS-CoV-2 infection remain relatively limited within the Romanian literature and clinical context. Although existing recommendations provide broader rehabilitation and chronic disease management guidance, structured outpatient assessment strategies adapted to heterogeneous chronic disease populations have been less frequently described [10,12]. Consequently, the observations generated by the present pilot study may contribute to future research exploring outpatient assessment strategies and multidisciplinary follow-up in patients with chronic diseases following SARS-CoV-2 infection.

### 4.1. Study Limitations

Because participants were recruited consecutively from a single tertiary-care outpatient center, selection bias cannot be excluded. Consequently, the findings may not be generalizable to broader populations of adults with chronic diseases assessed after SARS-CoV-2 infection. The heterogeneity of the included cohort should also be considered when interpreting the findings. Participants differed with respect to age, chronic disease profiles, and likely disease severity. Such heterogeneity reflects routine clinical practice because patients frequently present with multiple coexisting conditions; however, it limits disease-specific interpretation. Because disease-specific analyses were not feasible within the present pilot sample, larger studies involving stratified chronic disease cohorts will be necessary to determine whether different disease categories demonstrate distinct patterns of persistent symptoms and functional findings following SARS-CoV-2 infection.

Predominant circulating SARS-CoV-2 variants during the recruitment period were not systematically documented; therefore, potential differences in symptom persistence associated with viral evolution could not be evaluated. Information regarding acute COVID-19 severity, hospitalization duration, intensive care admission, oxygen supplementation, vaccination status, previous rehabilitation interventions, SARS-CoV-2 reinfections, and acute inflammatory severity indicators was not systematically available. Consequently, the potential influence of these factors on symptom persistence, post-effort desaturation, dyspnea severity, and overall functional findings could not be evaluated.

Because some participants were evaluated relatively early after infection, a proportion of the cohort may have represented prolonged post-acute recovery rather than established post-COVID condition according to WHO criteria. Additionally, the absence of a control group limits the ability to distinguish functional findings potentially associated with SARS-CoV-2 infection from the background symptom burden attributable to chronic diseases.

Furthermore, the exploratory categorization approach proposed in this study has not undergone external validation and should not be interpreted as a clinical decision-making tool. The descriptive categories were developed solely to facilitate interpretation of symptom burden and physiological findings within this pilot cohort. In addition, the threshold values used for the preliminary functional categories were selected pragmatically and have not been validated specifically in mixed chronic disease outpatient populations assessed after SARS-CoV-2 infection. Consequently, these cut-offs should not be interpreted as definitive clinical thresholds or severity criteria. In addition, several symptoms were recorded as patient-reported present/absent findings without the use of validated symptom-specific instruments; therefore, symptom severity and temporal evolution could not be evaluated.

Because the study was conducted in a real-world outpatient setting, the 1-min STS protocol allowed the use of armrests when necessary for safety and comfort. Consequently, test performance may not be directly comparable with protocols requiring participants to keep their arms crossed throughout the test.

### 4.2. Future Directions

Future investigations should focus on prospective longitudinal evaluation of symptom trajectories and functional outcomes, assessment of reproducibility between evaluators, and examination of clinically meaningful outcomes such as rehabilitation participation, symptom progression, healthcare utilization, and rehospitalization.

The inclusion of comparator groups and larger multicenter cohorts may help clarify the relationship between chronic disease burden and persistent functional findings following SARS-CoV-2 infection. Future studies may also determine whether simple outpatient assessment measures can contribute to reproducible and clinically useful approaches for describing functional status in patients with chronic diseases.

## 5. Conclusions

This exploratory pilot study describes symptoms and functional findings among adults with chronic diseases assessed after SARS-CoV-2 infection using simple outpatient clinical measures. Fatigue and dyspnea were the most frequently reported symptoms within the present cohort. These findings describe symptom patterns observed among adults with chronic diseases assessed after SARS-CoV-2 infection but should not be interpreted as evidence of causal attribution to the infection itself.

Simple assessment tools, including the mMRC dyspnea scale, Borg perceived post-effort scale, pulse oximetry, and the one-minute sit-to-stand test, may contribute to a more structured description of functional status in routine outpatient practice. The exploratory categorization approach presented in this study should be interpreted as hypothesis-generating and descriptive rather than as a validated clinical framework or decision-making model.

Given the exploratory design, small sample size, heterogeneous patient population, absence of a control group, and lack of longitudinal outcome assessment, the findings should be interpreted cautiously. Future prospective studies involving larger and preferably multicenter cohorts are needed to evaluate reproducibility, disease-specific patterns, and clinically meaningful outcomes in patients with chronic diseases assessed after SARS-CoV-2 infection.

## Figures and Tables

**Table 1:** Baseline characteristics of the study population (n = 38).

Characteristic	Value
Age (years), mean ± SD	64.2 ± 13.2
Age range (years)	38–82
Sex, n (%)	
Male	21 (55.3%)
Female	17 (44.7%)
Chronic comorbidities, n (%)	
Hypertension	23 (60.5%)
Ischemic heart disease	13 (34.2%)
Diabetes mellitus	9 (23.7%)
Chronic obstructive pulmonary disease	8 (21.1%)
Cancer	2 (5.3%)

Legend: Baseline demographic and clinical characteristics of the study population. Continuous variables are presented as mean ± standard deviation (SD), while categorical variables are expressed as number (n) and percentage (%). Percentages for chronic comorbidities may exceed 100% because some patients had multiple coexisting conditions.

**Table 2:** Distribution of Participants According to Time Since SARS-CoV-2 Infection (n = 38).

Time Since SARS-CoV-2 Infection	N	%
4 to <8 weeks	6	15.8
8 to <12 weeks	10	26.3
12 weeks to 6 months	22	57.9
Total	38	100.0

Legend: Distribution of study participants according to the interval between confirmed SARS-CoV-2 infection and outpatient functional assessment.

**Table 3:** Functional Assessment Findings (n = 38).

Variable	Value
mMRC dyspnea score, mean ± SD	1.1 ± 0.9
1-min sit-to-stand repetitions, mean ± SD	30.7 ± 4.1
1-min sit-to-stand repetitions, range	25–39
Borg score at rest, mean ± SD	0.8 ± 0.7
Borg score post-effort, mean ± SD	4.5 ± 1.6
SpO_2_ at rest (%), mean ± SD	96.0 ± 1.9
SpO_2_ post-effort (%), mean ± SD	93.2 ± 2.4
Heart rate at rest (bpm), mean ± SD	80.2 ± 10.4
Heart rate post-effort (bpm), mean ± SD	105.6 ± 12.7

Legend: Summary of functional assessment measures obtained during outpatient evaluation following SARS-CoV-2 infection. Values are presented as mean ± standard deviation (SD) unless otherwise specified.

**Table 4:** Descriptive Distribution of Severe Category Assignment According to Exploratory Classification Criteria (n = 13).

Driver of Severe Category Assignment	n (%)
mMRC ≥ 3 only	8 (21.1%)
Final post-effort SpO_2_ < 92% only	2 (5.3%)
Both criteria	3 (7.9%)
Total severe category	13 (34.2%)

Legend: Distribution of participants classified within the exploratory severe functional category according to the criterion responsible for category assignment. Percentages are presented relative to the total study population (n = 38).

**Table 5:** Descriptive Categorization of Functional Findings Observed During Outpatient Assessment.

Step	Clinical Component	Description/Criteria	Exploratory Interpretation
Step 1—Initial Screening	Inclusion criteria	Confirmed SARS-CoV-2 infection (≥4 weeks after acute phase) ≥1 chronic condition (hypertension, diabetes, COPD, ischemic heart disease, cancer)	Proceed to symptom assessment
Step 2—Symptom Assessment	Symptoms reported after SARS-CoV-2 infection	Fatigue Dyspnea Palpitations Sleep disturbances Anxiety	Document symptom burden
Step 3—Functional Assessment	Objective measures	mMRC dyspnea scale (0–4) Borg scale (rest and post-effort) Pulse oximetry (SpO_2_ at rest and after 1-min STS) Heart rate (rest and post-test)	Record functional findings
Step 4—Exploratory Functional Categorization	Combined interpretation	Mild: mMRC < 2 and ΔSpO_2_ < 4% (final SpO_2_ ≥ 94%) Moderate: mMRC ≥ 2 or ΔSpO_2_ ≥ 4% (final SpO_2_ ≥ 92%) Severe: mMRC ≥ 3 and/or final SpO_2_ < 92%	Descriptive categorization of functional findings
Step 5—Exploratory Observations Requiring Future Validation	Individualized assessment	Findings requiring further investigation in future studies	Exploratory observations requiring future validation in prospective studies.

Legend: The categorization approach presented in Table 5 was intended solely to describe functional findings observed during outpatient assessment. Referral decisions, rehabilitation pathways, and clinical outcomes were not evaluated within the present study. Consequently, the proposed categories should not be interpreted as guidance for rehabilitation referral or clinical decision-making but rather as exploratory observations requiring future validation.

## Data Availability

The data presented in this study are not publicly available due to ethical and legal considerations related to participant confidentiality. However, de-identified datasets may be made available from the corresponding author and principal investigator upon reasonable request.

## Abbreviations

The following abbreviations are used in this manuscript:

COVID-19	Coronavirus Disease 2019
SARS-CoV-2	Severe Acute Respiratory Syndrome Coronavirus 2
COPD	Chronic Obstructive Pulmonary Disease
mMRC	Modified Medical Research Council (dyspnea scale)
STS	Sit-to-Stand test
1-min STS	One-minute Sit-to-Stand test
SpO_2_	Peripheral capillary oxygen saturation
HR	Heart Rate
GDPR	General Data Protection Regulation
